# Formation and remodelling of septate junctions in the epidermis of isopod *Porcellioscaber* during development

**DOI:** 10.3897/zookeys.1101.78711

**Published:** 2022-05-18

**Authors:** Katja Kunčič, Polona Mrak, Nada Žnidaršič

**Affiliations:** 1 Department of Biology, Biotechnical Faculty, University of Ljubljana, Večna pot 111, 1000 Ljubljana, Slovenia University of Ljubljana Ljubljana Slovenia

**Keywords:** Crustacea, embryo, epithelia, junctional complex, morphogenesis, ultrastructure

## Abstract

Septate junctions (SJs) perform an occluding function in invertebrate epithelia and consist of parallel septa extending across the intercellular space between neighbouring cells. In addition, they are required for several morphogenetic processes in arthropods. The biogenesis of SJs during development is inadequately studied and it was characterised in detail only for various epithelia of *Drosophilamelanogaster*. This paper provides a detailed analysis of the ultrastructural differentiation of SJs in the epidermis of the terrestrial isopod *Porcellioscaber* during embryonic and postembryonic development. In this study, mid-stage embryo S13 was the earliest stage in which single septa were observed basally to the adherens junction (AJ). Differentiation of SJs during further development includes gradual elongation of septa arrays and formation of continuous arrays of septa. The enlargement of SJs in the epidermis is most pronounced at the transition from embryonic to postembryonic development and after the release of mancae from the marsupium. SJs of postmarsupial mancae are similar to those of adults, but are not yet as extensive. Comparison of the differentiation of SJs in the epidermis and hindgut of *P.scaber*, reveals a similar sequence of events. In addition, remodelling of SJs was observed in the epidermis of late marsupial mancae, the stage of cuticle renewal. Common features of SJs’ biogenesis in *P.scaber* and *D.melanogaster* ectodermal epithelia are indicated.

## Introduction

The epidermis functions as a protective barrier, as well as a sensory interface between an organism and the outer environment. Its apical surface faces the exterior and in arthropods is covered by a cuticle, which provides additional protection (Neville 1984; Compѐre et al. 2004; Dillaman et al. 2013). The epidermis of arthropods is a monolayered epithelium, whose cells are connected by adherens junctions (AJ) and septate junctions (SJ) (Tepass and Hartenstein 1994; Izumi and Furuse 2014; Jonusaite et al. 2015). AJs mainly provide mechanical stability to the tissue and SJs function as an occlusive barrier to paracellular transport and are thus involved in the regulation of transepithelial transport. In arthropod epithelia and in invertebrate epithelia in general, the AJs are located subapically. They are discernible as two electron-dense plaques on the cytoplasmic sides of neighbouring lateral cell membranes, separated by an intercellular space of constant width, which is filled with less dense homogenous material. In vertebrates, AJs are located basally to tight junctions, which occlude the space between neighbouring cells. In invertebrate epithelia, the occluding function is performed by SJs, which were first reported in two species of cnidarians by Wood (1959), and termed septate desmosomes. SJs consist of parallel septa which surround the cell circumferentially (Fristrom 1988; Tepass and Hartenstein 1994; Tepass et al. 2001; Izumi and Furuse 2014; Jonusaite et al. 2015). In sections that are perpendicular to the septa, their “ladder-like” ultrastructure is clearly discernible, as electron dense septa span an intercellular space of constant width. Distinct morphological variants of SJs have been described in different invertebrate species and in different epithelia (Izumi and Furuse 2014; Jonusaite et al. 2015). In arthropod epithelia, smooth and pleated SJs have been characterised (Flower and Filshie 1975; Noirot-Timothée et al. 1978; Tepass and Hartenstein 1994; Izumi and Furuse 2014; Jonusaite et al. 2015). Smooth SJs are present in endodermally derived epithelia and pleated SJs in ectodermally derived epithelia, including the epidermis and hindgut. Pleated SJs are located basally to the AJs. Structurally similar pleated SJs are a hallmark of mollusc epithelia, where they have been shown to differ in permeability in accordance with the physiological function of different epithelia. This has reinforced the concept that they have a versatile and dynamic role (Jonusaite et al. 2015).

Functions beyond the role of SJs as a diffusion barrier have been reported and involvement of SJ proteins in regulation of morphogenesis and in signal transduction pathways has been demonstrated (Lamb et al. 1998; Luschnig et al. 2006; Hall and Ward 2016; Lim et al. 2019; Rouka et al. 2020). Different SJ proteins have been shown to be essential for several morphogenetic processes (Hall and Ward 2016; Rouka et al. 2020). They are required for hindgut morphogenesis (Wells et al. 2013), morphogenesis of trachea (Bätz et al. 2014) and salivary glands (Hall and Ward 2016). The role of SJ proteins in cell shape changes during salivary glands morphogenesis has been suggested. Interestingly, many SJ protein mutants show defects in the architecture of cuticular structures. Delamination between epicuticle and procuticle was reported (Lamb et al. 1998) and irregular organisation in the tracheal taenidia (Wu et al. 2004) was observed. Data on the structural characteristics of the formation of intercellular junctions during development are very scarce and are mainly limited to studies of the model organism *Drosophilamelanogaster* (Tepass and Hartenstein 1994; Genova and Fehon 2003; Tiklová et al. 2010). Several studies have documented the key role of SJs for normal development of *Drosophila* (Tepass et al. 2001). Growing evidence also supports the view that SJ proteins are involved in a spectrum of developmental events in which their function is probably independent of their involvement in occluding establishment of SJs (Rice et al. 2021).

To advance the understanding of the biogenesis and function of SJs it is necessary to analyse the ultrastructural differentiation of SJs in different species and to compare the timing of major events in their biogenesis with the steps in the embryonic and postembryonic development of the organism. It is also advantageous to evaluate the biogenesis of SJs in different organs of the same species to identify common and/or tissue specific principles of the formation and function of SJs. In arthropods, the isopod crustacean *Porcellioscaber* Latreille, 1804 is a suitable species to address this issue as its embryonic and postembryonic development is well characterised by morphological staging systems (Wolff 2009; Milatovič et al. 2010). In addition, data on morphogenesis of the digestive system and the epidermis are available (Štrus et al. 2008; Mrak et al. 2014; Bogataj et al. 2019). Embryonic development and the first stages of postembryonic development of *P.scaber* take place in the aqueous environment of the marsupium, a brood pouch on the ventral side of the female. The early-stage embryos comprise stages S1-S5, mid-stage embryos stages S6-S15, and after hatching from the chorion, the outer egg envelope, embryos are termed late-stage embryos, through stages S16-S19. After release from the inner egg envelope, the vitelline membrane, marsupial mancae, including early-stage, mid-stage, and late-stage marsupial mancae develop within the marsupium for up to ten additional days (Milatovič et al. 2010; Mrak et al. 2012). During this early postembryonic development, the epidermis forms a new cuticle (Mrak et al. 2014). Postembryonic development proceeds after release from the marsupium as postmarsupial mancae stages, which are adapted to the external terrestrial environment (Tomescu and Craciun 1987). To the best of our knowledge, the ultrastructural differentiation of cell junctions in the epidermis of crustaceans has not been characterised to date. A recent study by Bogataj et al. (2019) provides a detailed ultrastructural analysis of the differentiation of SJs in the hindgut epithelium during development of *P.scaber*.

In this study we characterise the ultrastructural differentiation of SJs in the epidermal epithelium of *P.scaber* during embryonic and postembryonic development, based on transmission electron microscopy imaging and measurements of SJs’ structural characteristics. Our results are evaluated and discussed with respect to data on differentiation of SJs in the well-studied model organism *Drosophilamelanogaster* and with respect to SJ differentiation in the hindgut epithelium of *P.scaber*, aiming to unravel common features in the biogenesis of pleated SJs.

## Materials and methods

### Specimens of *Porcellioscaber*

Specimens of *P.scaber* Latreille, 1804 (Crustacea: Isopoda) were collected in Slovenia and placed in a glass terrarium with soil and leaf litter. Animals were maintained and bred at a constant temperature of 25 °C, high humidity and a 12 h light/dark cycle. Adult animals without a marsupium and without external signs of moulting were included in the analysis (Zidar et al. 1998). Embryos and marsupial mancae were isolated from the marsupia of gravid females. Embryonic developmental stages were characterised according to morphological characteristics defined in the developmental staging system established by Milatovič et al. (2010). Among 19 embryonic stages, the following stages were analysed in this study: five stages of mid-stage embryos (S10, S12, S13, S14, and S15) and two stages of late embryos (S16 and S18). The marsupial mancae stages were defined by morphological characteristics described in Mrak et al. (2012) as early-, mid-, and late-stage marsupial mancae. Postmarsupial mancae were collected three days or two weeks after release from the marsupium of the females which were reared individually in moist petri dishes. The images of specimens in different developmental stages were recorded with a MZFL III stereomicroscope (Leica) equipped with a Leica DFC425 C digital camera, using LAS V4.0 software.

### Sample preparation and imaging by light microscopy and transmission electron microscopy

Adult animals were anesthetised with diethyl ether before dissection. Tergites were isolated and cut along the median plane, fixed and decalcified overnight in a solution of 2% paraformaldehyde, 2.5% glutaraldehyde and 2.5% ethylenediaminetetraacetic acid (EDTA) in 0.1M HEPES buffer (pH 7.2). Intact embryos and mancae were fixed in 2.5% glutaraldehyde in 0.1 M cacodylate buffer (pH 7.2) at room temperature for 2 h and stored in the fixative at 4 °C for several days, needed to collect samples of different developmental stages. Egg envelopes surrounding the embryos were perforated with a thin needle or completely removed before fixation. Subsequent to fixation, all samples were rinsed with the same buffer that was used in the fixative and then postfixed for 2 h in 1% osmium tetroxide (OsO_4_). After rinsing with buffer, the specimens were dehydrated in ethanol, graded from 50% to 100%, transferred to pure acetone and finally infiltrated and embedded in Agar 100 epoxy resin. Prior to embedment, the surface of each manca was carefully perforated with a thin needle to improve infiltration of the resin. Resin polymerisation was performed at 60 °C for at least 24 h.

Semithin sections (0.5 µm) of the samples for light microscopy and ultrathin sections (~70 nm) for transmission electron microscopy were cut with a glass and a diamond knife respectively, using a Reichert Ultracut S ultramicrotome (Leica). The semithin sections were stained with Azure II – Methylene Blue (Richardson stain), dried and mounted in Ultrakitt (J.T. Baker) then inspected with an Axioscope Opton (Zeiss) light microscope. Micrographs of tergites and the dorsal body surface of embryos and mancae were obtained with a Leica DFC290HD digital camera using LAS V4.0 software. Ultrathin sections were contrasted for 10 min with uranyl acetate and for 5 min with lead citrate. They were analysed and imaged with a CM100 (Philips) transmission electron microscope, equipped with an Orius SC200 digital camera (Gatan) and Digital Micrograph software. Electron micrographs of tergites and the dorsal body surface of embryos and mancae were acquired and analysed.

### Measurements of structural characteristics of adherens and septate junctions

**Figure 1.**
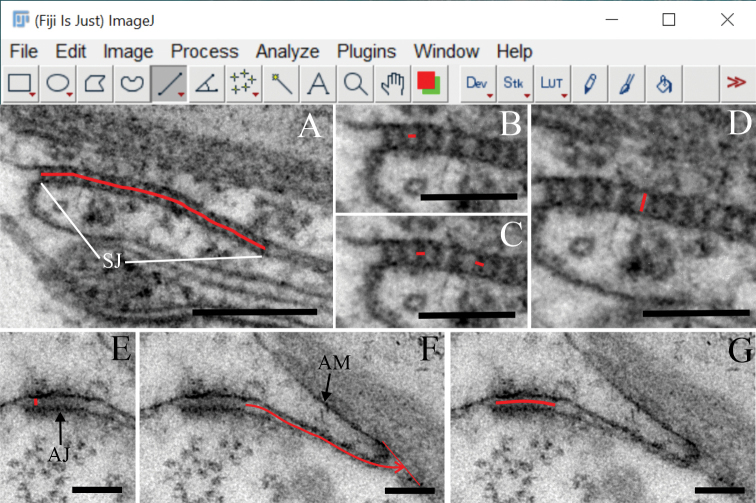
Measurements in ImageJ/Fiji **A–D** measurements of SJ characteristics **A** the length of a continuous array of septa was measured using the “segmented line” tool **B** the spacing between consecutive septa in an array is indicated **C** the thickness of septa is labelled **D** the width of intercellular space in the SJ region **E–G** measurements of AJs **E** the width of intercellular space in the AJ region **F** measurement of the distance of AJ from the apical membrane (red arrow) **G** measurement of the AJ length. Abbreviations: AJ: adherens junction; AM: apical membrane; SJ: septate junction. Scale bars: 200 nm (**A**); 100 nm (**B–G**).

Measurements of selected structural characteristics of cell junctions were performed using ImageJ/Fiji software on electron micrographs obtained in seven embryos (three mid-stage embryos S13 and four late-stage embryos S16), seven marsupial mancae (two early-, two mid-, three late-stage), in four postmarsupial mancae (postmarsupial mancae 3 or 14 days after release from marsupium, two of each) and in three adult animals. The following characteristics of the SJs and AJs were measured: (i) the length of a continuous array of septa, (ii) the spacing between consecutive septa in an array, (iii) the thickness of septa, (iv), the width of intercellular space in the SJ region, (v) the width of intercellular space in the AJ region, (vi) the distance of the AJ from the apical membrane and (vii) the length of the AJ (Fig. 1). In the measurement of the length of a continuous array of septa, the longest array of consecutive septa of each SJ was included, while subsequent or previous arrays that were shorter than the measured array, were not included. Measurements were carried out as follows. The length of a continuous array of septa was measured with the “segmented line” tool in the ImageJ/Fiji software, as shown in Fig. 1A. The spacing between consecutive septa in an array (Fig. 1B) and the thickness of septa (Fig. 1C) were measured with the “straight line” tool. The width of intercellular space was measured using the “straight line” tool as shown in Fig. 1D for SJs and as shown in Fig. 1E for AJs. The distance of the AJ from the apical membrane was measured with the “segmented line” tool in the Fiji software by lining the lateral membrane from the apical margin of the AJ to the apical membrane (Fig. 1F). The length of the AJ was measured using the “segmented line” tool (Fig. 1G).

To determine the statistically significant differences of junctions’ structural characteristics between groups the Kruskal-Wallis test was performed, followed by Mann-Whitney pairwise test with Bonferroni correction. In addition, to determine the statistically significant differences in the width of the intercellular space in the region of AJs compared to SJs, two sample Mann-Whitney tests were performed. Due to small sample sizes nonparametric tests were applied. All statistical tests were performed using PAST v4.03 software (Hammer et al. 2001; https://www.nhm.uio.no/english/research/infrastructure/past/). Data visualisation was performed by box-and-whiskers plots, generated using BoxPlotR, a web-tool for generation of box plots (Spitzer et al. 2014; http://shiny.chemgrid.org/boxplotr/). The edges of the box are the first (Q1) and the third (Q3) quartile, while the second quartile (Q2), the median, is represented by the line in the box. The whiskers represent the lowest and highest data within 1.5*IQR from the first and third quartile, respectively. Individual measurements are represented with dots.

### Scoring of the SJs’ architecture by semiquantitative criteria

In addition to the above measurements, we conducted an analysis of alterations in the architecture of SJs during development, using a scoring system of defined criteria. Five categories of SJ architecture were assigned accordingly: (i) single septa, (ii) short continuous array of septa (2–10 septa), (iii) discontinuous junctions containing short arrays, (iv) long continuous array of > 10 septa and (v) discontinuous junctions containing long arrays. Arrays of consecutive and regularly spaced septa were considered as continuous, while consecutive arrays of septa, separated by extended sections without visible septa, were considered as discontinuous. The following number of junctions were included in the semiquantitative evaluation: 13 junctions of mid-stage embryos (S13), 28 junctions of late-stage embryos (S16), 44 junctions of early marsupial mancae, 24 junctions of mid-stage marsupial mancae, 45 of late marsupial mancae, 55 and 33 junctions of postmarsupial mancae 3 days and 14 days after release from marsupium, respectively, and 20 junctions of adult animals.

## Results

### The ultrastructure of intercellular junctions in the epidermis of tergites in intermoult adult animals

**Figure 2.**
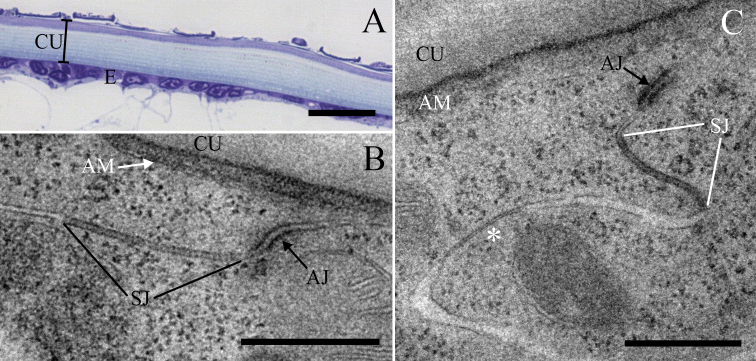
The ultrastructure of cell junctions in the epidermis of tergites of a *P.scaber* adult animal **A** semithin section of the tergite: The integument of adult animals consists of flattened epidermal cells covered by a thick cuticle **B** ultrastructure of an AJ and a pleated SJ with clearly resolved septa. SJs in adult animals are in the form of long continuous arrays of septa **C** an AJ in the subapical region of lateral cell membranes and a pleated SJ situated basally to the AJ. Further along the lateral membranes a close apposition of membranes is discernible (asterisk). Abbreviations: AJ: adherens junction; AM: apical membrane; CU: cuticle; E: epidermis; SJ: septate junction. Scale bars: 20 µm (**A**); 500 nm (**B, C**).

**Figure 3.**
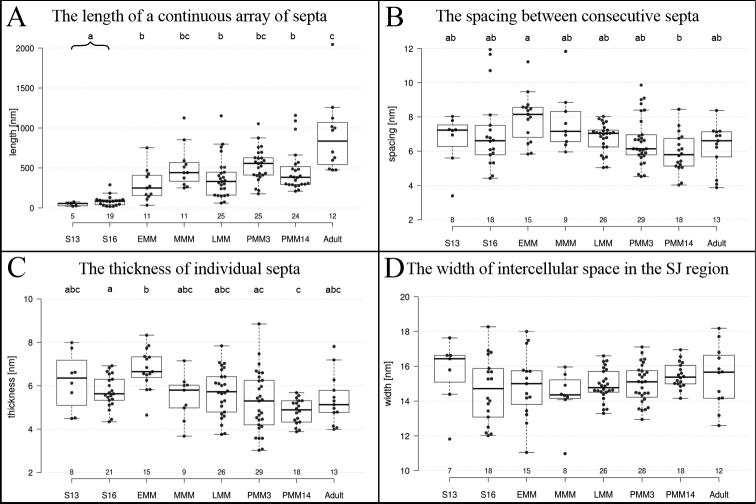
Measurements of the ultrastructural characteristics of SJs (**A–D**), graphically demonstrated by box-and-whiskers plots. Individual measurements are represented with dots and the numbers below the box-plots represent the number of measurements. The following stages were included in the analysis: mid-stage embryos S13 (S13), late-stage embryos S16 (S16), early-stage marsupial mancae (EMM), mid-stage marsupial mancae (MMM), late-stage marsupial mancae (LMM), postmarsupial mancae 3 days (PMM3), and 14 days (PMM14) after release from marsupium and adults. The letters above box-plots indicate significant differences between developmental stages (Mann-Whitney, *p* < 0.05). Measurements of the length of a continuous array of septa (**A**) in embryonic stages were pooled for statistical tests. Abbreviations: SJ: septate junction.

**Figure 4.**
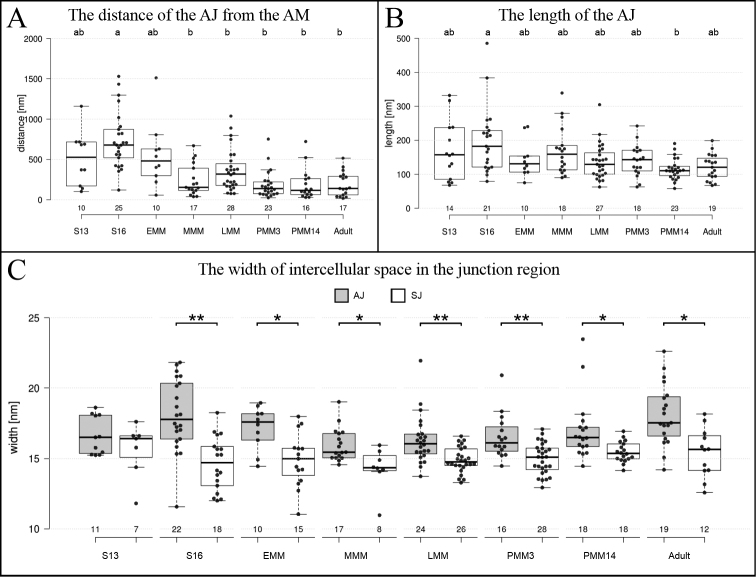
Measurements of the ultrastructural characteristics of AJs (**A, B**) and a comparison of the width of intercellular space in the AJs’ and SJs’ region (**C**), graphically demonstrated by box-and-whiskers plots. Individual measurements are represented with dots and the numbers below the box-plots represent the number of measurements. The following stages were included in the analysis: mid-stage embryos S13 (S13), late-stage embryos S16 (S16), early-stage marsupial mancae (EMM), mid-stage marsupial mancae (MMM), late-stage marsupial mancae (LMM), postmarsupial mancae 3 days (PMM3) and 14 days (PMM14) after release from marsupium and adults **A, B** the letters above box-plots indicate significant differences (Mann-Whitney, *p* < 0.05) between developmental stages **C** two sample Mann-Whitney tests were performed to determine statistically significant differences in the width of the AJs’ and SJs’ intercellular spaces of each developmental stage: *p* < 0.01 (*), *p* < 0.001 (**). Abbreviations: AJ: adherens junction; AM: apical membrane; SJ: septate junction.

The epidermis of tergites in intermoult adult animals consists of flattened epithelial cells covered with a thick cuticle (Fig. 2A). Lateral membranes of epidermal cells are connected with AJs and basally adjacent to them, pleated SJs (Fig. 2B, C). Ultrastructurally, SJs are clearly revealed as “ladders” of consecutive electron dense septa that span the intercellular space and connect electron dense lateral membranes (Fig. 2B). As evident in some sections lateral membranes basally to SJs were closely apposed (Fig. 2C). SJs were generally arranged in long continuous arrays of septa that measure ~ 840 nm (Figs 2B, 3A). Sequential arrays of septa, separated by intercellular space without septa, were also evidenced and are termed discontinuous junctions thereafter. When discontinuous junctions were present, intermediate regions without septa were predominately short. Septa were arranged in a regular pattern, approximately 7 nm apart from each other (Fig. 3B), and ~ 5 nm thick (Fig. 3C). The width of intercellular space in the junction region was approximately 16 nm (Fig. 3D). AJs were located subapically, above SJs, and at a variable distance, ~ 140 nm from the apical membrane (Figs 2B, 4A). The length of AJs was ~ 120 nm (Fig. 4B). Two electron dense plaques were seen on the cytoplasmic side of the adjacent cell membranes (Fig. 2B, C). The intercellular space in the AJ region was filled with material of intermediate electron density (Fig. 2B) and ~ 18 nm wide, which is significantly wider than the intercellular space of SJs (Fig. 4C).

### Early stages of SJ formation and the structure of AJs in the embryonic epidermis

**Figure 5.**
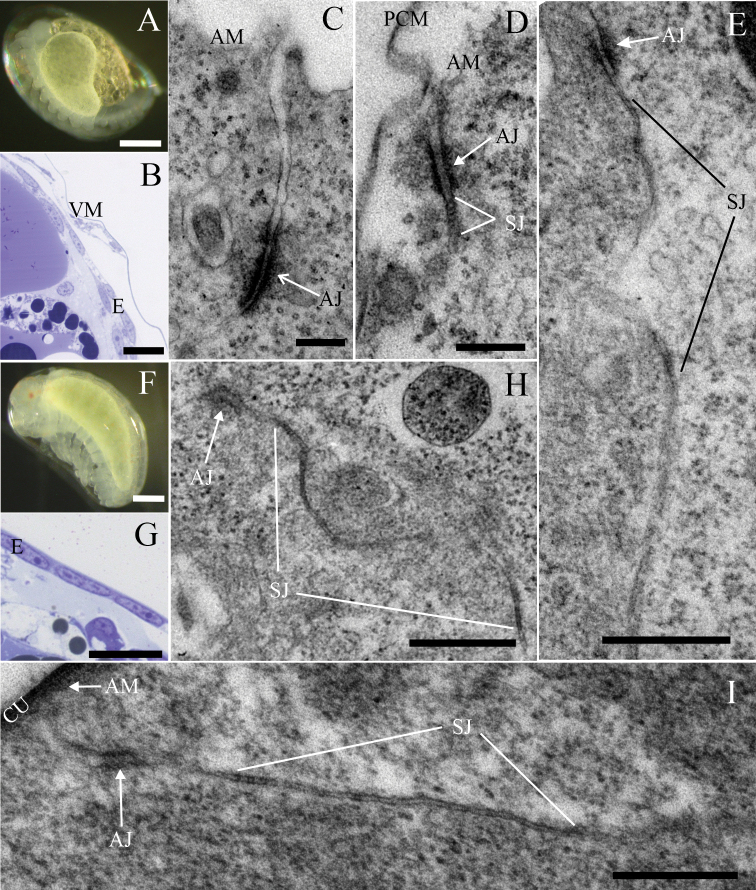
The ultrastructure of the dorsal body surface epidermal cell junctions in embryonic stages of *P.scaber***A–E** samples of mid-stage embryos **A** the external morphology of mid-stage embryo S13 **B** semithin section of an S13 embryo: Epidermal cells on the dorsal part of the body are flattened **C** in mid-stage embryos S10, an AJ is evidenced between neighbouring epidermal cells. Septa of SJs are not observed **D** mid-stage embryos S13: the AJ is located subapically and its ultrastructure is similar to that in adult AJs. A short array of septa is evident just beneath the AJ**E** mid-stage embryos S14: A discontinuous SJ containing short arrays of septa **F–I** samples of late-stage embryos **F** late-stage embryo S16 **G** semithin section of S16 embryo: Epidermal cells of dorsal body surface are flattened **H, I** in late-stage embryos S16 (**H**) and S18 (**I**) SJs are mainly discontinuous and consist of short arrays of septa. Abbreviations: AJ: adherens junction; AM: apical membrane; CU: cuticle; E: epidermis; PCM: precuticular matrix; SJ: septate junction; VM: vitelline membrane. Scale bars: 0,2 mm (**A, F**); 20 µm (**B, G**); 200 nm (**C, D**); 500 nm (**E, H, I**).

In this study, SJs with ultrastructurally discernible septa were first evidenced in the epidermis of mid-stage embryos (Fig. 5). While septa were not yet observed in mid-stage embryos of stage S10 (Fig. 5C) and S12, they were evidenced in S13 embryos (Fig. 5D) and in subsequent embryonic stages, including mid-stage embryos S14 (Fig. 5E) and late-stage embryos S16 (Fig. 5H) and S18 (Fig. 5I). Throughout these stages, we recorded single septa, continuous short arrays (Fig. 5D) and discontinuous junctions comprising short arrays of septa (Fig. 5E, H, I). The size of the longest continuous septa arrays was ~ 60 nm in mid-stage embryos S13 and ~ 80 nm in late-stage embryos S16, but this difference is not statistically significant (Fig. 3A). In mid-stage and late-stage embryos, the spacing between consecutive septa was ~ 7 nm and septa thickness ~ 6 nm (Fig. 3B, C). In mid-stage embryos S13 the median width of intercellular space in the SJs’ region was 16 nm and in the AJs’ region 17 nm, while in late-stage embryos S16 it was 15 nm in the SJs’ region and 18 nm in the AJs’ region. The comparison of the width of intercellular space in both junctional regions showed a significant difference for S16 (Fig. 4C). AJs were ubiquitous in the epidermal epithelia of all analysed embryonic stages of *P.scaber* and ultrastructurally similar to those in other analysed developmental stages and adult animals (Fig. 5C–E, I). AJs in embryonic stages were generally located more basally than in mancae and adults, ~ 530 nm from the apical cell surface in S13 and ~ 680 nm in S16 embryos (Figs 4A, 5C–E). The distance of AJs from the apical cell surface in embryos S16 is significantly larger in comparison to developmental stages from mid-stage marsupial mancae onwards (Fig. 4A). The length of AJs was ~ 160 nm in mid-stage embryos S13, and ~ 180 nm in late-stage embryos S16, which is larger than in all other stages and adults, but a significant difference is shown clearly between embryos S16 and postmarsupial mancae 14 days after release from marsupium (Fig. 4B).

### Formation of long arrays of septa and remodelling of SJs is characteristic for early postembryonic developmental stages

**Figure 6.**
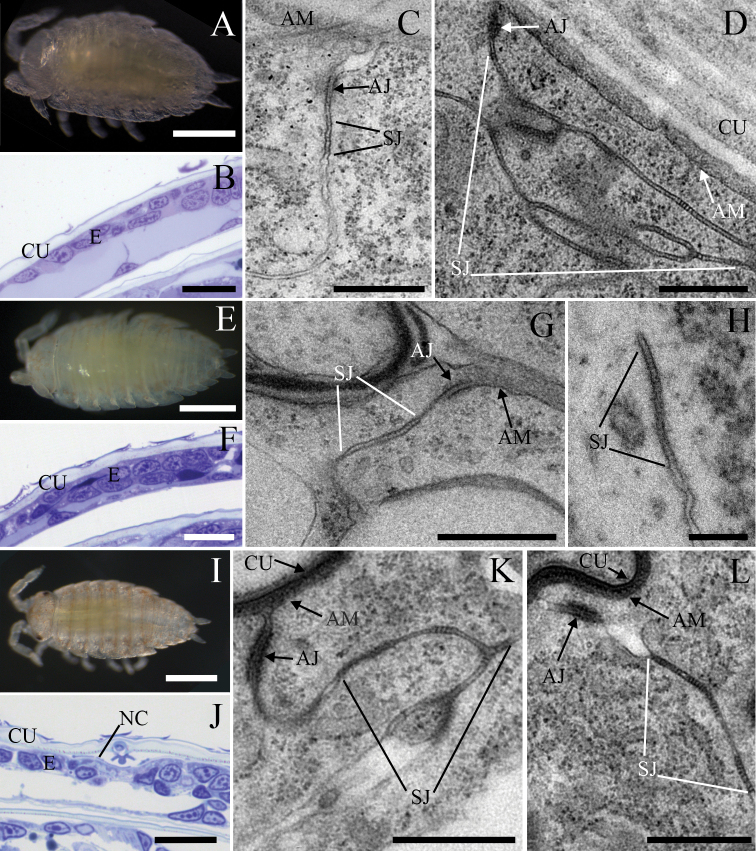
The ultrastructure of cell junctions between epidermal cells of tergites in marsupial manca stages of *P.scaber***A–D** samples of early marsupial mancae **A** early marsupial manca **B** semithin section of manca: epidermal cells of the tergite are flattened and covered by a cuticle **C** a short continuous array of septa is evident beneath the AJ**D** discontinuous junctions containing short arrays of septa are often evidenced along lateral cell membranes of neighbouring cells **E–H** samples of mid-stage marsupial mancae **E** mid-stage marsupial manca **F** semithin section of manca: The epidermis of the tergite consists of flattened cells, which are covered by a cuticle **G** the junctional complex consists of a subapically located AJ and basally adjacent to it a long continuous SJ**H** continuous long array of septa **I–L** samples of late marsupial mancae **I** late marsupial manca **J** semithin section of late manca epidermis: Epidermal cells are not as flat as in all other analysed developmental stages. Detachment of the cuticle and formation of a new cuticle reveal the renewal of the exoskeleton **K** discontinuous junctions containing short arrays of septa are often evidenced beneath the AJ in late marsupial manca stage **L** discontinuous long arrays of septa are rarely observed in late-stage marsupial mancae. Abbreviations: AJ: adherens junction; AM: apical membrane; CU: cuticle; E: epidermis; NC: new cuticle; SJ: septate junction. Scale bars: 500 µm (**A, E, I**); 20 µm (**B, F, J**); 500 nm, (**C, D, G, K, L**); 200 nm (**H**).

We analysed the ultrastructure of AJs and SJs in the epidermis of marsupial manca stages (Fig. 6) to evaluate the differentiation of junctions after hatching from the vitelline membrane, i.e. at the beginning of the postembryonic development (early marsupial mancae) and at the stage of cuticle renewal (late marsupial mancae). Early-stage marsupial mancae, immediately after hatching from the vitelline membrane (Fig. 6A–D), was the only marsupial manca stage in which single septa were noted, however short continuous and discontinuous arrays of septa prevailed (Fig. 6C, D). In mid-stage marsupial mancae epidermis (Fig. 6E–H), the most common were long continuous arrays of septa (Fig. 6G, H), while short continuous and discontinuous arrays were less frequent. Interestingly, in late-stage marsupial mancae (Fig. 6I–L) discontinuous junctions, mainly those containing short arrays, were most often observed (Fig. 6K, L). A change in cell shape was also indicated in this stage, as cells were more cuboidal in contrast to flattened cells observed in previous and subsequent developmental stages (Fig. 6J). Data on the length of a continuous array of septa show a statistically significant increase in marsupial manca stages in comparison to embryos (Fig. 3A). The length of continuous arrays of septa was ~ 250 nm in early-, ~ 440 nm in mid- and ~ 330 nm in late-stage marsupial mancae (Fig. 3A). The spacing between consecutive septa was ~ 8 nm in early and ~ 7 nm in late and mid-stage marsupial mancae (Fig. 3B). Septa thickness was ~ 7 nm in early- and ~ 6 nm in mid- and late-stage marsupial mancae (Fig. 3C). The width of the intercellular space at the SJs’ site was ~ 15 nm in early and late marsupial manca stages and ~ 14 nm in mid-stage marsupial mancae (Fig. 3D). The intercellular space within the AJs was ~ 18 nm in early-stage, ~ 15 nm in mid-stage and ~ 16 nm in late-stage marsupial mancae, in all stages significantly wider than the intercellular space within SJs (Fig. 4C). The location of AJs was generally subapical, ~ 480 nm from the apical membrane in early-, ~ 150 nm in mid- and ~ 320 nm in late-stage marsupial mancae (Figs 4A, 6C, G, K, L). In comparison to late embryos S16, the AJs of mid- and late-stage marsupial mancae are located closer to the apical cell surface (Fig. 4A). The size of AJs was ~ 130 nm in early, ~ 160 nm in mid and ~ 130 nm in late marsupial manca stage (Fig. 4B).

### Continuous and discontinuous long arrays of septa are characteristic for SJs in postmarsupial manca stages

**Figure 7.**
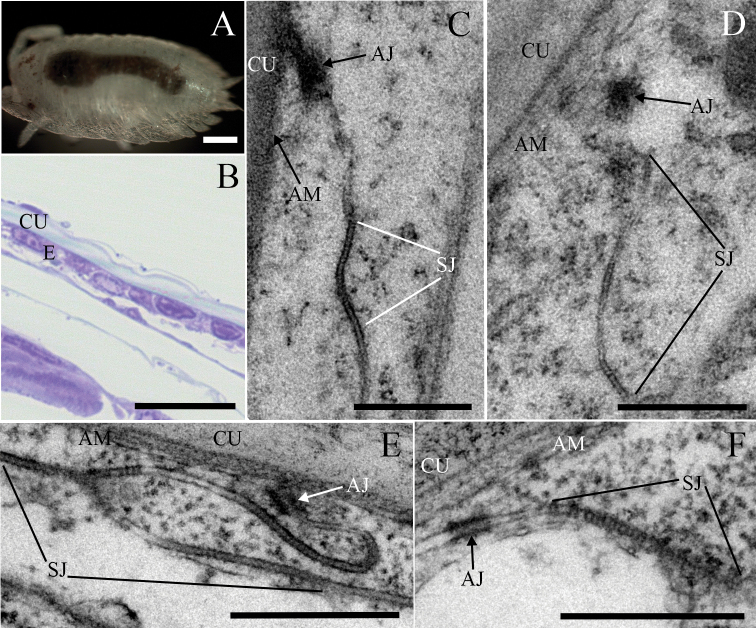
The ultrastructure of cell junctions in the epidermis of tergites in *P.scaber* postmarsupial mancae **A** external morphology of postmarsupial manca **B** semithin section of epidermis: Flattened epidermal cells are covered by a cuticle that is not yet as thick as in adult animals **C–D** AJs and SJs of postmarsupial mancae 3 days after release from marsupium **C** a long continuous array of septa is evident between neighbouring cells **D** discontinuous junctions containing short arrays of septa are rarely observed **E–F** epidermal cell junctions of postmarsupial mancae 14 days after release from marsupium **E** discontinuous junction containing long arrays encompasses the lateral membranes **F** long continuous SJs are often observed. Abbreviations: AJ: adherens junction; AM: apical membrane; C: cuticle; E: epidermis; SJ: septate junction. Scale bars: 200 µm (**A**); 20 µm (**B**); 500 nm (**C–F**).

We analysed samples of postmarsupial mancae to evaluate later stages in the differentiation of SJs and the effect of the change of environment, from the marsupium to the external environment (Fig. 7). Postmarsupial mancae 3 and 14 days after release from marsupium were analysed. SJs were predominately evidenced as long continuous arrays or discontinuous junctions containing long arrays and an increase in abundance of septa in comparison to previous developmental stages was observed (Figs 3A, 7C, E, F). The length of continuous arrays of septa was ~ 560 nm in mancae 3 days after release from marsupium and ~ 380 nm in mancae 14 days after release from the marsupium (Fig. 3A). Septa were ~ 6 nm apart (Fig. 3B) and ~ 5 nm thick (Fig. 3C) in both postmarsupial manca stages. The width of the intercellular space in the SJs’ region was approximately 15 nm, and significantly wider in the AJs’ region, ~ 16–17 nm (Fig. 4C). The AJs were ~ 140 nm and ~ 120 nm from the apical membrane in postmarsupial mancae 3 days and 14 days after release from marsupium, respectively (Fig. 4A). The approximate length of AJs was 140 nm in postmarsupial mancae 3 days after release from marsupium and 110 nm in postmarsupial mancae 14 days after release from marsupium (Figs 4B, 7E, F).

**Figure 8.**
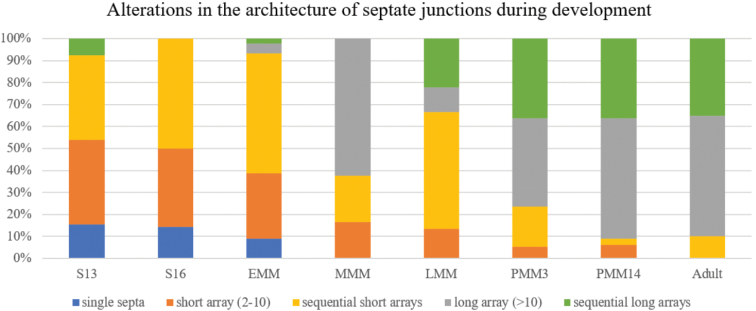
Semiquantitative analysis of the alterations in the architecture of SJs during development. The analysis includes mid-stage embryos S13 (S13), late-stage embryos S16 (S16), early-stage marsupial mancae (EMM), mid-stage marsupial mancae (MMM), late-stage marsupial mancae (LMM), postmarsupial mancae three days after release from the marsupium (PMM3), postmarsupial mancae 14 days after release from marsupium (PMM14), and adult animals.

A summary of the alterations in the architecture of SJs in the epidermis of *P.scaber* throughout development is presented according to our semiquantitative analysis (Fig. 8). SJs are first established in mid-stage embryos S13 and are evidenced as single septa and short continuous or discontinuous arrays of septa. The established SJ architecture is maintained through embryonic stages. In early postembryonic development SJs gradually expand, until they are generally configured as long continuous arrays of septa in mid-stage marsupial mancae. The stage of late marsupial manca reintroduces the predominance of discontinuous junctions containing short arrays of septa. This remodelling of SJs coincides with exoskeletal cuticle renewal in late marsupial mancae. SJs in postmarsupial manca stages exhibit predominately continuous long arrays of septa or discontinuous junctions containing long arrays of septa. In the epidermis of intermoult adults SJs are generally comprised of long continuous arrays of septa or discontinuous junctions containing long arrays of septa, while single septa and short arrays of septa were not detected.

## Discussion

### The ultrastructure of SJs in *P.scaber* tergite epidermis

The ultrastructure of SJs in tergite epidermis of intermoult adult *P.scaber* was characterised and compared with SJs in the hindgut epithelium of the same species (Bogataj et al. 2018) and with pleated SJs in different ectodermal epithelia of adult specimens of other arthropod species (Happ and Happ 1970; Lane and Skaer 1980; Noirot-Timothée and Noirot 1980).

The epidermal and hindgut epithelia of *P.scaber* are both monolayered, ectodermal in origin and covered by a chitinous cuticle, but they have several morphological and ultrastructural specialisations reflecting their different functions. Epidermal cells are flattened and covered by a thick and mineralised cuticle, which forms a protective barrier (Hild et al. 2008; Seidl and Ziegler 2012). On the other hand, hindgut epithelial cells are isodiametric and covered by a cuticle that is not mineralised. The hindgut cuticle is ten times thinner than the exoskeletal cuticle and displays a less pronounced organisation of lamellae, a pattern appearing due to helicoidal arrangement of chitin-protein fibres (Bogataj et al. 2018). Lateral cell membranes in the epidermis are slightly convoluted, however they are intensely interdigitated in the hindgut epithelium. In addition, in the hindgut the apical and basal membrane form membrane labyrinths, which are associated with numerous mitochondria in the papillate region (Bogataj et al. 2018). These ultrastructural features of the hindgut epithelium are in accordance with the transporting and osmoregulatory role of the two functional regions of the hindgut, the anterior chamber and papillate region, respectively (Hryniewiecka-Szyfter and Storch 1986; Hames and Hopkin 1989; Bogataj et al. 2018). Comparative ultrastructure of pleated SJs in the epidermis and hindgut epithelium of *P.scaber* shows considerable differences in the architecture of SJs. In comparison to the mainly long continuous SJs in the epidermis, SJs in the hindgut epithelium are even more extensive, mainly discontinuous and intensely convoluted due to the interdigitations of lateral plasma membranes. Such interdigitations are more pronounced in the papillate region of the hindgut (Bogataj et al. 2018). In the hindgut, several dilations of the intercellular space were evident regularly in between the septal arrays (Bogataj et al. 2018). In the epidermis, the dilations of the intercellular space are not as numerous, however they can span over larger areas below the SJ. In the epidermis, the intercellular space of characteristic width for SJs was filled in some cases with finely granulated material of medium electron density and septa were not clearly resolved. Another distinction in the ultrastructure is related to microtubules in the vicinity of SJs. Bogataj et al. (2018) reported an abundance of microtubules in the vicinity of SJs in both regions of the hindgut, but we did not observe this in the epidermis. The role of the hindgut epithelium in transport and osmoregulation suggests a pronounced need for the restriction of paracellular transport (Hryniewiecka-Szyfter and Storch 1986; Hames and Hopkin 1989) and the differences in the architecture of SJs in the epidermis and in the hindgut epithelia, are likely related to the functional differences between both ectodermal epithelia.

Pleated SJs in the tergite epidermis of adult intermoult *P.scaber* characterised in this study consist of long continuous or discontinuous arrays of electron dense septa. Discontinuous pleated SJs have already been described in arthropods by Noirot-Timothée and Noirot (1980). Ziegler (1997) studied the ultrastructure of the sternal epithelium in *P.scaber* and reported also on the apical cell contacts between cells. Cell junctions of sternal epithelial cells comprise AJs, SJs and additional cell contacts, characterised by a reduced distance between plasma membranes. Subsequent research by Ziegler and Merz (1999) has confirmed these contacts as gap junctions. We report a similar architecture of apical cell junctions in the *P.scaber* tergite epidermis, and we observed the locations of apposition of plasma membranes basally to SJs, which are similar to gap junctions described in Ziegler (1997).

A general width of 15 nm of the intercellular space in the region of SJs, as described here for the epidermis of *P.scaber*, has previously been reported for pleated SJs in arthropods (Lane and Skaer 1980; Noirot-Timothée and Noirot 1980). Regularly spaced septa, ~ 6 nm apart, are representative for SJs in the epidermis of *P.scaber*. While this regularity has also been reported in the epidermis of different arthropods (Lane and Skaer 1980; Noirot-Timothée and Noirot 1980), a study by Lane and Skaer (1980) has shown that septa in different tissues of the locust *Schistocercagregaria* are either regularly or irregularly spaced. A spacing between septa of pleated SJs has been reported to be in the range of 16–20 nm in different arthropod epithelia (Happ and Happ 1970; Lane and Skaer 1980; Noirot-Timothée and Noirot 1980). Noirot-Timothée and Noirot (1980) defined that the reported spacing of septa in their study is attributed to the center-to-center spacing, however other authors did not specify the method of measurements. In our study, the septal distance was specified as the space between the edges of consecutive septa. Distinct methods of measurements could contribute to the difference of septal distance between our results and data from other studies. Our results in *P.scaber* tergite epidermis have revealed an average septal thickness of 5 nm, however for other arthropods, a septal thickness of 2–9 nm has been reported (Lane and Skaer 1980; Noirot-Timothée and Noirot 1980). These results indicate that septal thickness and spacing in pleated SJs varies considerably in different arthropod epithelia, while the width of intercellular space in SJs’ region is rather invariable.

In addition to SJs, epidermal cells of *P.scaber* are circumferentially surrounded and connected by subapically located AJs. Our analysis of AJs in the tergite epidermis of adult specimens of intermoult *P.scaber* has shown a ubiquitous presence as well as a uniform ultrastructural appearance of two regularly spaced electron dense plaques on the cytoplasmic side of lateral plasma membranes. Similar results have previously been reported by Bogataj et al. (2018) in epithelia of both hindgut regions of the same species. In our analysis, we have also shown a variability in the length of individual AJs and in the location with regard to the apical membrane.

### The formation of SJs in the epidermis of *P.scaber* during embryonic and postembryonic development involves a gradual increase in the abundance of the septa and the formation of continuous arrays

A detailed study of ultrastructural differentiation of pleated SJs in arthropod epithelia has been performed in the common fruit fly, *Drosophilamelanogaster* by Tepass and Hartenstein (1994). The molecular composition of SJs in the fruit fly has been characterised in several studies (Izumi and Furuse 2014; Hall and Ward 2016; Rice et al. 2021). The biogenesis of SJs in the hindgut epithelium of *P.scaber* was characterised by Bogataj et al. 2019. Only fragmentary data on the formation of SJs, referring mainly to specific developmental stages are available for other arthropod species (Locke 1965; Hagopian 1970; Caveney and Podgorski 1975; Noirot-Timothée and Noirot 1980; Lane and Swales 1982). We show in this paper that the ultrastructural differentiation of SJs in the epidermis of *P.scaber* during embryonic and postembryonic development is characterised by consecutive steps of junction assembly, beginning with the formation of a few electron-dense septa near the AJs in mid-embryonic stage S13. Differentiation of SJs during further development includes gradual elongation of septa arrays and formation of continuous arrays of septa. A gradual increase of the extent of SJs during late embryonic and postembryonic development was evidenced also in the hindgut epithelium of *P.scaber* (Bogataj et al. 2019). A similar sequence of events in SJs’ biogenesis has been reported in different epithelia of *D.melanogaster*, including the epidermis, trachea, pharynx, oesophagus, inner layer of proventriculus, hindgut and salivary glands (Tepass and Hartenstein 1994; Tepass et al. 2001). Taken together, these results suggest a common principle of SJs’ biogenesis in *D.melanogaster* and *P.scaber* ectodermal epithelia, but there are considerable differences in the timelines of SJs’ formation in these two species. In *D.melanogaster*, SJs’ biogenesis occurs in the range of several hours, while in *P.scaber* the duration of these events is measured in days. The core SJ proteins are expressed early in *D.melanogaster* embryonic development and are localised along the lateral membrane by stage 12, after roughly 9 hours of embryogenesis (Hall and Ward 2016; Rice et al. 2021). In fruit fly embryos that have reached stage 14, the first septa have been identified (Tepass and Hartenstein 1994; Rice et al. 2021). Gradually, more septa are formed, and studies of functionality have shown that the paracellular barrier is established in stage 15, after 12 hours of embryonic development (Paul et al. 2003; Rice et al. 2021). In the next 10–12 hours regularly arranged septa are formed and are described as a hallmark of stage 17 embryos, the final stage of *D.melanogaster* embryonic development (Rice et al. 2021). Mature SJs are attributed to the first instar larva of *D.melanogaster* (Tepass and Hartenstein 1994), but further postembryonic development was not followed in this respect (Tepass and Hartenstein 1994; Rice et al. 2021). In *P.scaber* epidermis, initial septa are formed by approximately the 17^th^ day of embryogenesis and additional septa are added throughout embryonic development. When the embryo hatches from the vitelline membrane (on ~ 25^th^ day of embryogenesis) and the epidermis is thereafter exposed to the marsupial fluid, the arrays of septa become more abundant. During postembryonic development of marsupial mancae, SJs are further elongated due to additional septa incorporation and long arrays of septa are as frequent as short arrays. Approximately on the 35^th^ day of development, mancae are released from the marsupium to the external environment and the architecture of SJs resembles that in adult animals, but the septa are still not as abundant as in adults.

Our results show that the intercellular space in the region of SJs was not significantly different in analysed developmental stages. As for the thickness and spacing of consecutive septa, our analysis did not reveal a clear pattern of changes corresponding to developmental stages. The main difference between immature and mature SJs is in the abundance and distribution of septa. To the best of our knowledge there are no other reports on measurements of SJs’ structural characteristics during SJs’ biogenesis in relation to tissue morphogenesis. There are however some studies which offer fragmentary data on SJs in specific developmental stages of various arthropodal species (Locke 1965; Hagopian 1970; Caveney and Podgorski 1975).

The remodelling of SJs is characteristic for *P.scaber* late marsupial mancae epidermis (shown in this study) and for the hindgut epithelium (Bogataj et al. 2019). SJs’ remodelling in the hindgut epithelium is more conspicuous and is characterised by a considerable shortening of the junction, while in the epidermis continuous junctions are transformed into discontinuous and shorter arrays of septa. The main physiological process in late marsupial manca stage that could be associated with SJs’ remodelling in analysed ectodermal epithelia of *P.scaber* is cuticle formation. However, for the hindgut, Bogataj et al. (2019) have also suggested a possible contribution of the feeding pause in moulting mancae to SJs’ remodelling. In general, data on the remodelling of SJs on the ultrastructural level are sparse. Reversible remodelling of pleated SJs was examined by Khan and Saleuddin (1981) in the renal epithelium of adult gastropod *Hellisoma* (Mollusca: Gastropoda), and the changes were attributed to experimental exposure of animals to a hypoosmotic medium. In the tracheal epithelium of *D.melanogaster*, a link between the formation of the chitin matrix and SJs has been established, identifying specifically the role of SJ proteins in the secretion of chitin deacetylases Serp and Verm (Wang et al. 2006; Luschnig et al. 2006; Nelson et al. 2010). Deacetylases Serp and Verm are required to form and modify a chitin cylinder that serves as a template for accurate tracheal morphogenesis, but their absence in *serp* and *verm* mutants has no effect on the barrier function of tracheal epithelia (Luschnig et al. 2006). In addition, Serp and Verm as well as SJ components are required for the correct formation and the rigidity of the epidermal cuticle. The absence of Serp and Verm affects the shape of the body. The study by Lamb et al. (1998) also showed that correct SJs’ formation in *D.melanogaster* is essential for the structure of embryonic cuticle. The absence of Coracle, a structural protein of SJs, affects the localisation of other SJs’ components and the ultrastructure of SJs. A consequence of Coracle absence is that the embryonic cuticle is thinner and the epicuticle fails to adhere to the procuticle, resulting in two detached layers (Lamb et al. 1998). It is known that epidermal cell shape changes during moulting cycle in crustaceans (Compѐre et al. 2004; Dillaman et al. 2013), and it was also indicated in this study that the height of the epidermis is enlarged in late marsupial mancae. It seems likely that SJs' remodelling in late marsupial mancae accompanies cell shape changes.

Our measurements indicate a decrease in the distance of AJs from the apical membrane at the transition to postembryonic development while results on the length did not show significant changes in relation to development. For *D.melanogaster*, Tepass and Hartenstein (1994) showed that AJs in ectodermal epithelia are fully formed shortly after gastrulation and that their ultrastructure does not significantly change throughout the development. Tepass and Hartenstein (1994) reported a generally 20–60 nm long band of AJs in the larval epidermis of *D.melanogaster*. The constant ultrastructure of the AJs is consistent with their role in the establishment of cell polarity, formation of apical and basolateral plasma membrane domains, and maintenance of tissue integrity (Tepass et al. 2001; Payre 2004).

## Conclusions

Pleated SJs in the tergite epidermis of adult intermoult *P.scaber* characterised in this study consist of long continuous or discontinuous arrays of electron dense septa and are less extensive than in the hindgut epithelium of the same species. We consider that distinct ultrastructures of SJs reflect different functions of both epithelia and suggest also differences in the paracellular barriers.

We determined the first stage of septa formation in the epidermis of mid-stage embryo S13, where single septa and short arrays of septa were detected. Further formation of SJs in the epidermis of *P.scaber* during embryonic and postembryonic development involves a gradual increase in the abundance of the septa and the formation of continuous arrays. The enlargement of SJs in the epidermis is most pronounced at the transition from embryonic to postembryonic development and after the release of mancae from the marsupium. A similar sequence of SJs’ biogenesis has also been reported in the hindgut of the same species. The subsequent addition of septa until long arrays of septa are formed appears to be representative of SJs’ biogenesis in the ectodermal epithelia of arthropods.

The late marsupial manca stage represents a period of SJs’ remodelling and conversion of continuous junctions to discontinuous and shorter arrays. Similar, but more pronounced remodelling of SJs was described in the hindgut epithelia of *P.scaber* in the same developmental stage. We consider that these changes in SJs’ architecture in the analysed ectodermal epithelia of *P.scaber*, are related to the processes of moulting.
